# An open‐source software for monitoring intrafraction motion during external beam radiation therapy based on superimposition of contours of projected ROIs on cine‐MV images

**DOI:** 10.1002/acm2.12940

**Published:** 2020-06-07

**Authors:** Rémi Lessard, Nicolas M. Tremblay, Marc‐Émile Plourde, Mathieu Guillot

**Affiliations:** ^1^ Département de radio‐oncologie Centre hospitalier universitaire de Sherbrooke (CHUS) Sherbrooke Québec J1H 5N4 Canada; ^2^ Faculté de médecine et des sciences de la santé Université de Sherbrooke Sherbrooke Québec J1H 5N4 Canada; ^3^ Département de médecine nucléaire et radiobiologie Université de Sherbrooke Sherbrooke Québec J1H 5N4 Canada

**Keywords:** cine‐MV, intrafraction motion management, open‐source software, projection

## Abstract

**Purpose:**

To present an open‐source software (https://github.com/CHUSRadOncPhys/FluoMV) for monitoring intrafraction motion that is based on the visualization of superimposed contours of projected region‐of‐interests from DICOM RTSTRUCT files on cine‐MV images acquired and displayed in real‐time during radiation therapy delivery. Clinical use with prostate gold fiducial markers is presented.

**Methods:**

Projections of regions of interest (ROI) in the reference frame of the electronic portal imaging device are computed offline for different gantry angles before the first treatment fraction. During treatment delivery, the contrast of portal images is automatically adjusted using a histogram equalization algorithm. The projections associated with the current gantry angle are then superimposed on the images in real time. This allows the therapist to evaluate if the imaged structures of interest remain within their respective contours during treatment delivery and to potentially interrupt the treatment if deemed necessary. The spatial accuracy of the method was evaluated by imaging a ball bearing phantom in a set‐up where the position of the projected ROI is highly sensitive to gantry angle errors. The visibility of fiducial markers during one fraction of seven different volumetric modulated arc therapy (VMAT) prostate treatments is characterized.

**Results:**

The geometric validation showed a negligible systematic error μ < 0.1 mm for the position of the projections. The random errors associated with the time accuracy of the gantry angle readout were characterized by standard deviations σ ≤ 0.6 mm. The VMAT clinical treatments showed that the fiducial markers were frequently visible, allowing for a meaningful clinical use.

**Conclusions:**

The results demonstrate that the method presented is sufficiently accurate to be used for intrafraction monitoring of patients. The fact that this method could be implemented on many modern linacs at little to no cost and with no additional dose delivered to the patients makes this solution very attractive for improving patient care and safety in radiation therapy.

## 1 INTRODUCTION

Modern linac‐based radiation therapy uses imaging to accurately position target volumes. For example, medical linacs are now equipped with MV and kV imaging systems as standard and techniques such as portal imaging, kV radiography, or cone‐beam computed tomography (CBCT) are typically used at the beginning of each treatment fraction to minimize interfraction positioning errors. During treatment delivery, intrafraction positioning errors may occur due to internal organ or patient motion. Margins are usually applied to the target volume during treatment planning to account for these effects. Motion management systems can also be used to monitor the patient position during treatments. Available solutions include optical surface imaging,^1^ Varian RPM,^2^ MV,^3^ and kV^4^, ^5^ imaging with or without fiducial markers, magnetic resonance imaging (MRI),^6^ ultrasound imaging,^7^ and radiofrequency (RF) implants.^8^ Those systems often include an automatic beam‐gating functionality that can start and stop the radiation based on the position of the target volume relative to predefined thresholds.

The intrafraction monitoring systems mentioned above have one or more disadvantages. First and foremost, the costs associated with additional hardware, such as optical, ultrasound, and RF implants monitoring systems, can be a disincentive, while integrated magnetic resonance (MR)‐Linac solutions are simply out of reach for most radiation therapy centers because of their high costs. Kilovoltage imaging techniques give additional radiation doses to the patient which may pose a health risk, especially when many fractions are delivered.^9^ Another drawback of many real‐time kV imaging systems is that the images are acquired in a plane orthogonal to the treatment beam. Therefore, it is not possible to guarantee that the beam does not miss the target, as it would be the case if the images were acquired in the beam's eye view geometry. As for the use of fiducial markers and RF implants, it requires additional invasive medical procedures with associated risks. Lastly, optical monitoring techniques of the patient surface are unable to detect errors related to internal organ movements, such as the displacement of the prostate due to gas movement in the rectum or bladder filling.

Cine‐MV imaging has been the subject of many publications.^10^, ^11^, ^12^ In most of those, the target volume is tracked by detecting fiducial markers or image features. However, few of them have implemented solutions that use contours defined during treatment planning to evaluate the intrafraction motion of structures of interest,^13^ as is done with other imaging modalities^14^, ^15^ and for the evaluation of interfraction positioning with DRRs^16^ and CBCT.^4^, ^17^

The purpose of this work is to present an open‐source software that uses superimposition of contours of projected regions of interests (ROIs) on cine‐MV images to monitor intrafraction motion of patients with Elekta linacs (Elekta Limited). A validation of its spatial accuracy and a presentation of its clinical use with patients treated for prostate cancer using volumetric modulated arc therapy (VMAT) with implanted gold fiducial markers are also presented. By making our software open source, we hope to give clinicians a useful tool to assess the accuracy of intrafraction positioning of patients in their clinics. The software can be downloaded on GitHub (https://github.com/CHUSRadOncPhys/FluoMV) and instructions for installation and use are provided.

## 2 MATERIALS AND METHODS

The software was designed for Elekta linacs equipped with a PerkinElmer electronic portal imaging device (EPID) and has been tested on Synergy and Infinity linac models. The software includes a ROI projection module that can be run on a quad‐core CPU or on a NVIDIA graphics processing unit (GPU) for faster computation, and an acquisition module that runs on the computer connected to the EPID. The EPID is an amorphous silicon detector panel (XRD 1640 AL5 P) that produces images of 1024 × 1024 pixels with a digital resolution of 16 bits per pixel. The EPID is located at a distance of 160 cm from the radiation source and has a field of view of 25.6 cm × 25.6 cm at the linac isocenter.

### 2.A ROI projection module

The projections are computed from the ROIs defined by the radiation oncologist or the planner in a treatment planning system (TPS) and exported as a DICOM RTSTRUCT file. To compute the two‐dimensional projections of a three‐dimensional (3D) ROI, the linac geometry and the coordinates of the plan isocenter provided by the DICOM RTPLAN file are used. The first step of the projection algorithm is to create a 3D voxel grid with coordinates that matches those of the planning CT images. This grid contains binary values, where all voxels have a value of 0 except those inside the ROI to be projected. Secondly, for a given gantry angle and for each group of 2 × 2 detector elements of the EPID,^1^ the equation parameters of the straight line passing by the position of the pixel and the position of the radiation source are determined. Then, for each X, Y, and Z plane of the grid that contain at least one voxel with a value of 1, the coordinates of the point of intersection of the plane with the line are calculated and rounded to the nearest integer to find in which voxel the intersection occurs. As soon as an intersection occurs in a voxel with a nonzero value, the computation for this EPID detector element is stopped and a value of 1 is set to its corresponding pixel on a two‐dimensional (2D) binary map that represents the EPID image. If this condition is never met for a given detector element, the corresponding pixel in the binary map is set to 0. The contours of the projected ROI are then generated by selecting only the pixels of the binary projection map with a value of 1 that have at least one of their eight neighbors with a value of zero or that is located on a boundary of the map.

For a given patient, this projection process is done only once, before the first fraction of the treatment, for every desired ROI, at intervals of 0.5° over 360°. The data are saved in a file accessible by the computer that controls the EPID. At the time of treatment, the therapists use the graphical user interface to enter the patient identification number, select the prescription, and the contours they want to visualize during treatment delivery. Finally, the projected contours are superimposed on the live MV EPID images during treatment delivery. Since the projections are precomputed, the time efficiency of the projection algorithm has no effect on the real‐time display of the contours during the treatments.

### 2.B Acquisition modules

The software used to visualize the clinical treatments is installed on the Elekta iView computer located in the control room of the linear accelerator. The software includes two acquisitions modules that are executed in parallel on different processor threads: the image acquisition module and the iCom messages acquisition module. The image acquisition module uses the *XISL.dll* dynamic link library provided by the EPID manufacturer to directly control the detector and read in real time the frame buffer of the EPID frame grabber board. The acquisition mode is set to *Free running* which corresponds to the situation where the panel sends out images continuously to the frame grabber at a frequency equal to the inverse of the image integration time. The integration time is set to the shortest time available, 433 ms, and a timestamp corresponding to the time when the panel readout is completed is assigned to each image. The timestamp is based on the QueryPerformanceCounter function of the Microsoft Windows API and has a resolution < *1 μs*.

The iCom acquisition module uses the dynamic link library *iCOMClient.dll* provided by the linac manufacturer to read the iCom messages sent continuously by the linac. Information on the linac state, gantry angle, dose rate, and the nominal beam energy is acquired at a rate of approximately 4 Hz^18^ and a timestamp is assigned to each message at the time it is received. The linac states are used as trigger to start and stop image acquisition. During acquisitions, the image, the nominal beam energy, and the gantry angle interpolated at the image timestamp are continuously sent to the image processing and display module.

### 2.C Image processing and display module

Image processing has three purposes: (a) to produce images with minimal artifacts by calibrating the pixels response, (b) to produce images that are registered in the same reference frame as the precomputed ROI projections, and (c) to adjust the image contrast to better differentiate the structures. The pre‐processing steps consist of a subtraction of a dark frame acquired prior to irradiation, followed by a pixel gain correction, an image translation, a spatial median filtering, and a pixel binning. The pixel gain calibration file and the panel position correction table used for image translation are loaded in memory at the opening of the software and are specific to each linac. The gain calibration file consists of flood field images acquired at different dose rates for a given nominal beam energy. The panel position correction table contains the average displacements of the panel in the AB and GT directions^2^ relative to the central beam axis as a function of the gantry angle (see Section 2.D. for more details). From this table and from the interpolated gantry angle at the image timestamp, a translation is performed on each image so that the central beam axis coincides with the center of the image, as it is assumed in the ROI projection module. The spatial median filtering is performed for noise elimination purposes. To be consistent with the spatial resolution of the ROI projection module, the original images of size 1024 × 1024 are converted into a size of 512 × 512 by performing a 2 × 2 pixel binning.

Once the preprocessing of the image is completed, histogram equalization is performed on pixels of value superior to a threshold in order to obtain a good contrast in the radiation‐field region and thus be able to differentiate the structures of interest. By default, this threshold is 70% of the maximum gray‐level value of the image but can be adjusted during the delivery with a scroll bar if the contrast is not deemed good enough. Pixels below this threshold are given a value of 0 and all the others have their gray‐level value redistributed between 0 and 255. Once the contrast is adjusted, the precomputed projected ROIs are superimposed using the same colors specified in the TPS to be easily recognized. In addition, a checkbox list of the names of the ROIs is included in the software to allow users to choose whether or not to display any ROI at any time during the treatment. The whole process of image preprocessing, contrast adjustment, and superimposition of contours is sufficiently fast to be executed between two consecutive image readouts.

To determine which projection is to be displayed on the image, the gantry angle at which the image was acquired must be known. As a first approximation, a gantry angle can be assigned to each image by linearly interpolating the gantry angles of the iCom messages to the timestamp of the image. However, this method neglects the effect of the time order of the readout of the columns of the panel detector elements which can be significant when the ROI projected contours are off‐centre and the gantry rotation speed is high. Physically, the panel is divided laterally (in the AB direction) into two independent sections that have their own electronic reading system. The columns of the panel are parallel to the GT axis. The two sections are read synchronously, column by column, from the outside to the inside of the panel.^19^, ^20^ Therefore, for the section on the linac A‐side, the readout starts at column c=0 at t=0 and ends at column c=511 at t=433  ms. For the linac B‐side section, the readout starts at column c=1023 at t=0 and ends at column c=512 at t=433  ms. The exact readout time $t\left(c\right)$ of a column is as follows:

(1) $$t\left(c\right)=TS-0.433+\alpha \left(c\right)\times \frac{0.433}{511}$$

where *TS* is the timestamp of the image, and $\alpha \left(c\right)=c$ when $c\in \left[0,511\right]$ and $\alpha \left(c\right)=1023-c$ when $c\in \left[512,1023\right]$. For each ROI, we choose to approximate the image acquisition time as the readout time of the column corresponding to the center of the ROI projection in the image: *x_c_*. The temporal correspondence between an image and the iCom messages is therefore an iterative process that depends on *x_c_*, which itself depends on the gantry angle. For each new image acquired, the first step consists of linearly interpolate the gantry angles of the iCom messages to the timestamp *TS* of the image. Then, the coordinate of the centroid of the ROI projection for this angle is read and Eq. (1) is used to determine a new timestamp that corresponds to the readout of the pixel column that matches the ROI centroid coordinates. The gantry angle of the iCom messages are then interpolated to this new timestamp *t(c)* and the ROI projection corresponding to this new gantry angle is superimposed on the EPID image.

### 2.D Validation of spatial accuracy of ROIs projections

**Fig. 1:**
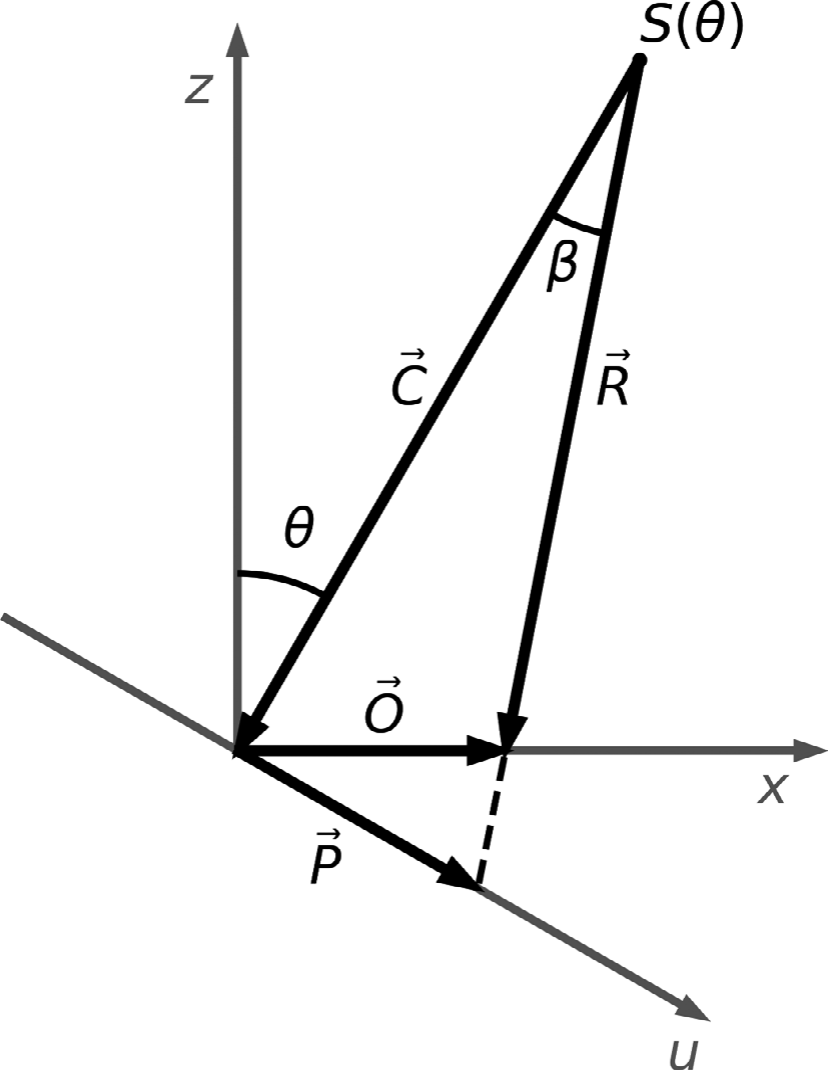
The x and z axes represent the lateral and vertical axes in the treatment room and the u axis represents the projection axis at isocenter in the X‐Z plane. The linac isocenter is located at the origin. When the gantry angle is θ, the radiation source is at position $S\left(\theta \right)$. The projection $\vec{P}$ of an object located at $\vec{O}$ can be calculated by determining the vectors $\vec{C}$ and $\vec{R}$ and deducing the angle β.

The accuracy of the position of the projected ROIs on the EPID images depends on many factors. The resolution of the treatment planning images on which the contours are initially defined, the angular resolution of the precomputed projections, and the resolution of the EPID images on which the ROIs are projected are fundamental limitations of the method. Other factors can affect the accuracy of the projections, such as the accuracy of the gantry angles associated with the images. Geometrically, gantry angle errors have an effect mainly in the AB direction. The magnitude of this effect depends on the position of the object relative to the radiation source. A geometrical analysis of the accuracy of the positions of the projected ROIs in the AB direction for different gantry angle errors and for different object positions is presented in Section 3.A. Figure 1 illustrates the geometry used for the analysis. When the gantry angle is θ, the radiation source is at position $S\left(\theta \right)=\left(SAD\cdot \sin\theta ,SAD\cdot \cos\theta \right)$ in the X‐Z plane, where *SAD* is the source‐to‐axis distance. The projection of the object located at $\vec{O}=\left(O_{x},0\right)$ can be found by calculating the vector:

(2) $$\vec{P}\left(\theta \right)=∥\vec{C}∥\cdot \tan\beta \cdot \hat{u}$$

where $\hat{u}$ is the unit vector along the u axis and:

(3) $$\beta =\pm \cos^{-1}\left(\frac{\vec{C}\cdot \vec{R}}{∥\vec{C}∥∥\vec{R}∥}\right)$$

The angle β is positive when $-90^{\circ }\leq \theta \leq 90^{\circ }$ and negative otherwise.

The accuracy of the projections also depends on the reproducibility of the panel position with respect to the gantry angle. To characterize this effect, an 8‐mm‐diameter radio‐opaque ball bearing (BB) phantom was fixed to the treatment table and positioned at the linac radiation isocenter. The EPID was then deployed at a gantry angle of 0°. Two 360° arc beams of 1000 monitor units with a field size of 10 cm × 10 cm were irradiated in the clockwise and the counter‐clockwise direction, respectively. This sequence was repeated five times producing ten measurements total given the two rotation directions. During irradiation, acquisitions were performed using the EPID acquisition module and the iCom acquisition module to assign a gantry angle to each image. Finally, the pixel coordinates of the center of the BB was found for each image which is by definition the central position of the panel in the ROI projection module. The results are presented in the Section 3.B. An average curve was obtained from the ten measurements and a panel position correction table was generated from that curve. The panel position correction table was defined as the offset in pixels to bring the center of the BB to the center of the image for every gantry angle.

**Fig. 2:**
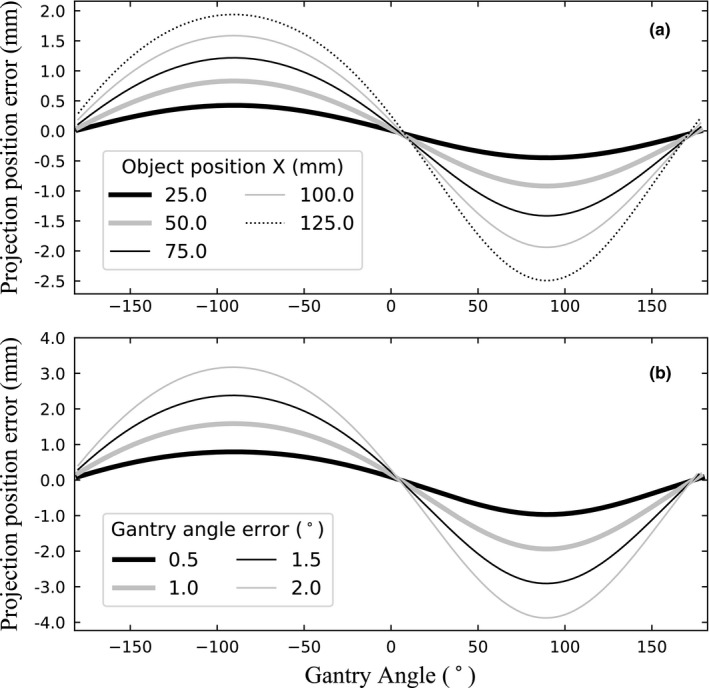
Simulation of the position errors of the projections in the AB direction caused by: (a) a systematic gantry angle error of 1° for different positions of an object and (b) different systematic gantry angle errors for an object located at X = 10 cm. In all cases, Y = 0 cm and Z = 0 cm. The EPID pixel size used was 0.25 mm.

To evaluate the overall accuracy of the method, an end‐to‐end test was performed using a BB phantom. First, a CT scan of the phantom was obtained with a transverse resolution of 0.5 mm × 0.5 mm and a slice thickness of 1.0 mm. It was then imported in a treatment planning system. A treatment plan was generated with two 360° clockwise arc beams of fixed field size of 24 cm × 24 cm. The monitor units were adjusted in order to have a constant angular speed of 1.5°/s for the first arc beam and 4.8°/s for the second arc beam, which correspond, respectively, to the minimum and maximum gantry speed of the VMAT plans in our clinic. The plan isocenter was positioned with an offset of 10 cm relative to the center of the BB in the lateral direction and with an offset of 5 cm in the longitudinal direction. In theory, this configuration is one of the most sensitive to gantry angle errors, mainly because of the lateral shift, as shown in Fig. 2. Then, the BB was contoured in the treatment planning system. The DICOM images, structures, and plan were exported and the projections were precomputed with the ROI projection module. Finally, during delivery, the calibrated EPID images were saved. The projection errors were assessed retrospectively by computing the difference between the centroid of the BB and the centroid of the contours. The results are presented in Section 3.C.

### 2.E Clinical use cases

To demonstrate the usefulness of the method in clinical situations, images were acquired with the software during VMAT treatments of seven patients treated for prostate cancer. The patients already had three gold fiducial markers (3 mm long, 1 mm diameter) implanted in the prostate for pretreatment CBCT image guidance. The distribution of the periods of visibility of the fiducial markers in the images was then generated. The planning CT images had a transverse resolution of 1.2 mm × 1.2 mm and a slice thickness of 3 mm. All the patients were treated with 6 MV dual arc VMAT treatment plans with a dose of 2 Gy per fraction. The plans were generated with the TPS Monaco version 5.11.02. The ROIs of the fiducial markers were manually drawn in the treatment planning system by the planners and a 3D isotropic expansion of 5 mm of those ROIs was performed to be used as tolerance margins for intrafraction motion. All the patients were positioned using CBCT as pretreatment image guidance. The registration of the CBCT images was done with the planning CT images by matching the fiducial markers. The default threshold setting for the histograms equalization was used in all cases. Results are presented in Section 3.D.

## 3 RESULTS

### 3.A Simulation of the impacts of gantry angle errors

Figure 2(a) shows, for a systematic gantry angle error of 1°, the position error of the projections in the AB direction as a function of the gantry angle for an object located at different lateral positions. The axes (*X,Y,Z*) correspond to the lateral, longitudinal, and vertical axis in the reference frame of the treatment room with the linac isocenter as the origin. According to Fig. 1, the projection position error in the AB direction is defined as:

(4) $$P_{\mathrm{error}}=\vec{P}\left(\theta +\varepsilon \right)-\vec{P}\left(\theta \right)$$

where ε is the gantry angle error. The results show that the projection error increases with increasing lateral distance of the object from the isocenter. For objects located at the edge of the panel, a gantry angle error as low as 1° can cause an error of up to 2.5 mm in the projections. Figure 2(b) shows the position error of the projections in the AB direction of an object located at 10 cm from the isocenter as a function of the gantry angle for different systematic gantry angle errors. The projection position error in the AB direction increases rapidly with the gantry angle error which explains why an interpolation of the gantry angle between the iCom messages has been deemed necessary (see Section 2.C).

On Figs. 2(a) and 2(b), the change of sign of the error coincides with the change of the direction of the displacement of the object in the image as the gantry angle changes.

Therefore, if the case simulated was a clockwise arc with a systematic positive gantry angle error, the projection error would always represents a contour that is ahead in time relative to the actual position of the object, even though the sign of the projection error changes. However, the same figure could also represent a contour that lags behind the actual position of the object if the arc was counter‐clockwise with a positive systematic gantry angle error.

### 3.B Panel position reproducibility

**Fig. 3:**
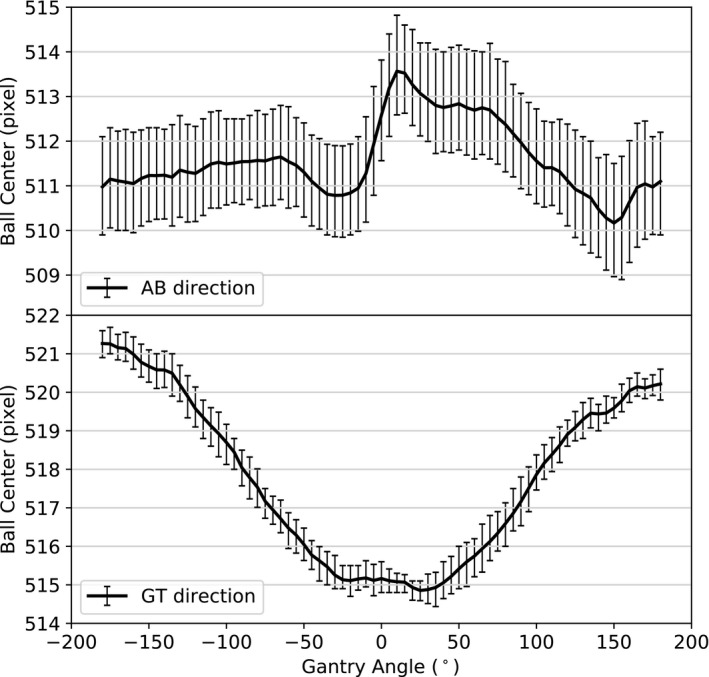
Average coordinates of the center of the BB as a function of the gantry angle before the application of the panel position correction table. The error bars represent the maximum deviations. For clarity purposes, the gantry angles were sampled at intervals of 5° on the figure.

Figure 3 shows, in the panel reference frame, the average pixel coordinate and maximum deviation of the position of the center of the BB as a function of the gantry angle for the ten measurements before the application of the panel position correction table (Section 2.D). The reproducibility of the panel position at any given gantry angle was mostly within ± one pixel (0.25 mm at the isocenter) for both AB and GT directions. The mean maximum deviation was 0.28 mm in the AB direction and 0.11 mm in the GT direction, and the ranges of deviations were [−0.35 mm: +0.37 mm] and [−0.16 mm: +0.17 mm], respectively. These results indicate that the contribution of the panel position error to the total error is inferior to one pixel on the final image which has a resolution of 0.5 mm after the pixel binning process. Consequently, once the panel position correction table is applied, the panel position is sufficiently reproducible to allow accurate registration of the images in the coordinate systems used for the projection computation. From Fig. 3, the panel position correction table can be obtained by determining the translation to be performed so that the center of the BB coincides with the center of the image [pixel coordinate (511.5, 511.5)] for each gantry angle.

### 3.C ROI projections accuracy on a phantom

**Fig. 4:**
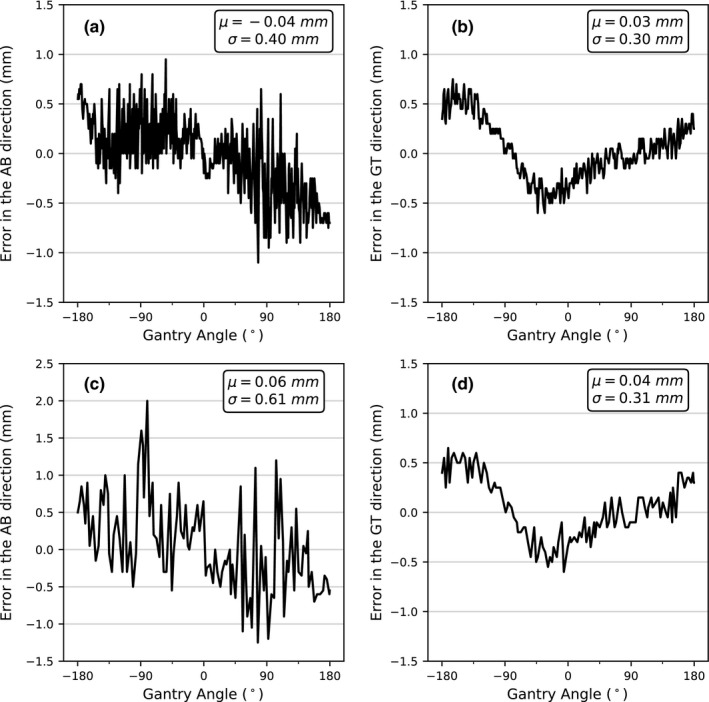
Position errors of the projections measured in the AB and GT directions for a clockwise rotation with gantry angular speed of 1.5°/s [(a) and (b)] and 4.8°/s [(c) and (d)]. Contrary to Fig. 2, a negative error always indicates that the contour lags behind the BB and a positive error always indicates that the contour is ahead of the BB.

Figure 4 shows the difference between the position of the projected contour of the BB and the real position of the BB in the images as a function of the gantry angle for the two gantry angular speeds tested. As mentioned in Section 2.D, the BB was positioned with an offset of 10 cm in the lateral direction and 5 cm in the longitudinal direction relative to the isocenter. As expected, the errors predominantly occurred in the AB direction. The error in the GT direction is <1 pixel and is minimally affected by the gantry angle speed. Contrary to Fig. 2, we have inverted the polarity of the errors in the AB direction for the positive gantry angles so that a negative error always indicates that the contour lags behind the BB and that a positive error always indicates that the contour is ahead of the BB. This allows the average value of the errors to be used to identify a systematic gantry angle error which may be caused by a bad calibration of the encoders or a systematic timing mismatch between the images and the iCom messages. For the gantry angular speed of 1.5°/s and 4.8°/s, the average errors are µ = −0.04 mm and µ = 0.06 mm with standard deviations of σ = 0.40 mm and σ = 0.61 mm, respectively, indicating that the gantry angle error has a very small systematic component and is mainly random. The results also show that the standard deviation of the error is larger for the arc with a greater angular speed. The source of the randomness has been identified as the temporal imprecision of the iCom messages sent by the linac (see Section 4). The causes of the residual error have not been identified but the error is of the order of one pixel (0.5 mm) which is small compared to the tolerance margins we use in our clinic for intrafraction motion (5 mm). Overall, the results demonstrate that the method is highly accurate despite the combination of all the possible errors, even in the extreme condition of an object located 10 cm off‐axis in the lateral direction.

### 3.D Clinical use

**Fig. 5:**
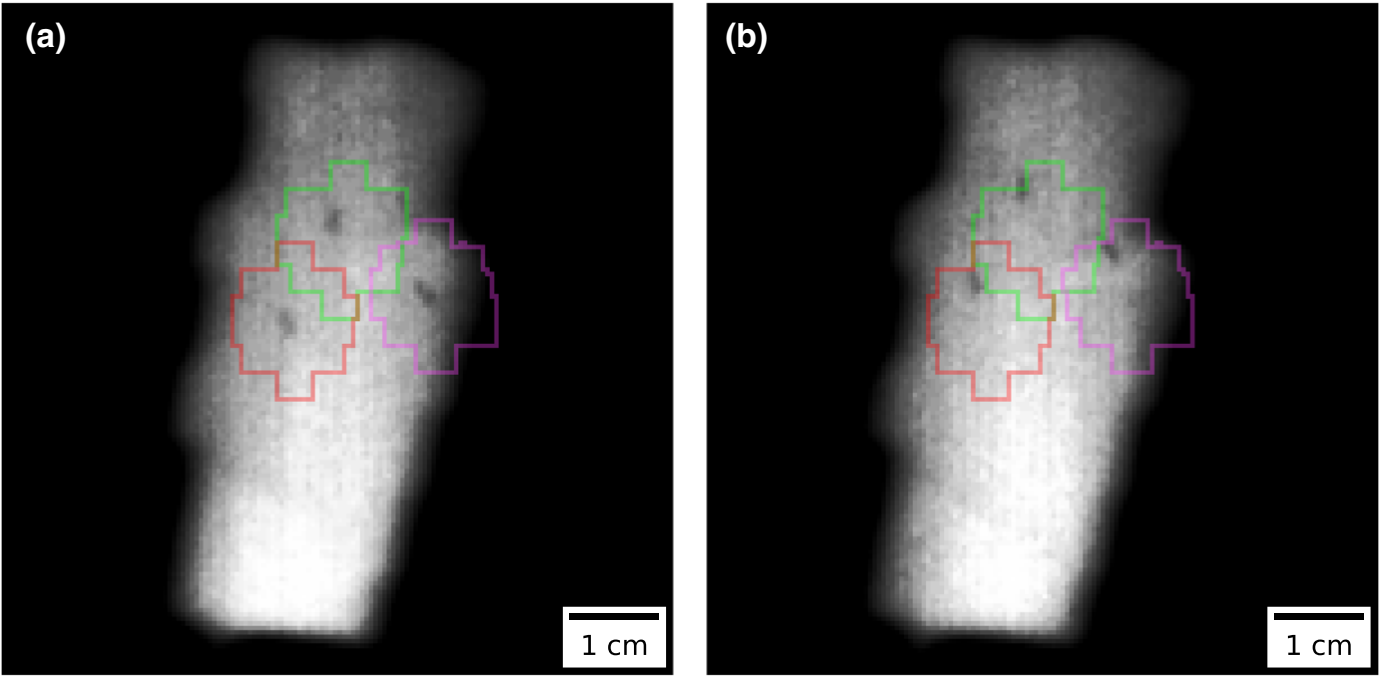
Example of images acquired at the same gantry angle during two different fractions of a volumetric modulated arc therapy treatment. The superimposed contours are the projections of a 3D isotropic expansion of 5 mm of the fiducial markers ROIs. These contours are used as tolerance for the movements of the markers. In (a) the fiducial markers are inside the tolerance contours. In (b) an intrafraction movement occurred and the therapist decided to interrupt the treatment because the markers are on the edge of our institutional tolerance margin of 5 mm.

**Fig. 6:**
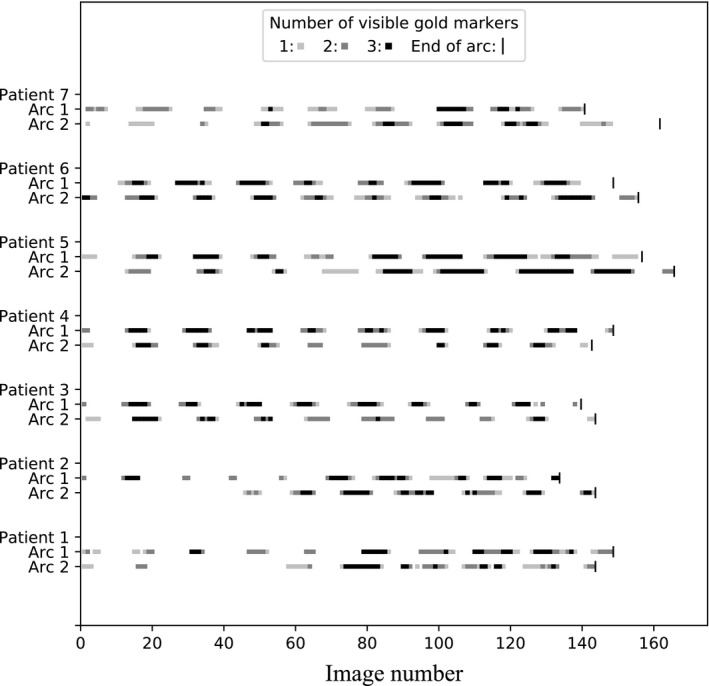
Number of visible fiducial markers on each image acquired during the course of volumetric modulated arc therapy treatments.

An example of two images acquired during VMAT treatments is presented in Fig. 5. Figure 6 shows the number of fiducial markers that could be seen on each image acquired continuously during one fraction for seven patients. The results demonstrate that despite the presence of some images with poor contrast and the fact that VMAT fields have dynamic aperture shapes where the leaves can hide the markers, their visibility occur frequently and with duration of a few seconds throughout the course of arc delivery. The average frequency at which the markers could be seen for all arcs of the seven patients is presented in Table 1, where the frequency is defined as the inverse of the elapsed time between the first image frame of two consecutive periods of time with continuous visibility of markers. This frequency can be used to estimate the number of spot checks that can be made on the position of the markers during beam delivery. Table 1 also presents the mean duration of those periods of time as well as the average fraction of time of the treatments where the markers are visible. Results are presented without making any differentiation between the markers; only the number of visible markers was used.

**Table 1:** Mean values of frequency, duration, and fraction of treatment of the fiducial markers visibility computed for the 14 arcs shown in Fig. 6.

	Frequency (1/s)	Duration (s)	Fraction of treatment
At least one marker	1/6.5	3.1	0.46
At least two markers	1/6.8	2.3	0.34
All three markers	1/8.5	1.6	0.18

## 4 DISCUSSION

The method for intrafraction monitoring presented in this work has some advantages over the other techniques discussed in the introduction. Most importantly is that the software is freely available and the technique does not require any additional hardware not already available on Elekta linacs equipped with an EPID model XRD 1640. The software source code is available under a MIT license and instructions for installing and using the software are provided. The technique also has the advantage of not giving additional dose of radiation to patients since the treatment beam is used for imaging.

For clinics interested in using the software, we expect clinical implementation to be done without major difficulty by following the instructions provided in the project repository. At the moment, the software has been tested on Synergy and Infinity Elekta linac models. The actual version of the software takes a lot of time for the calculation of projections if it is executed on a CPU, so it is highly recommended to get a GPU and use the GPU version for a routine clinical use. The software should be compatible with all treatment planning systems because only DICOM files are used. Once the pixel gain and panel position calibrations are completed, we recommend that the user carry out the commissioning test presented in section 3.C with a BB phantom. Different gantry speeds should be used for the two directions of rotation. It is not necessary to place the BB at an extreme lateral position as in section 3.C. More realistic clinical situations, such as the BB offset laterally but still closer to the isocenter, could be more relevant in determining the clinical accuracy of the method.

It is known that the poor contrast of MV images compared to kV images limits the capability to differentiate the different structures present in radiological images. This is especially true for limited field of view like those of collimated treatment beams where there can be a lack of anatomical landmarks to help identify which structures are present in the images. However, when used with gold fiducial markers and with proper contrast enhancement techniques, the contrast is generally good enough to discern the markers in the image as shown in Figs. 5 and 6. Moreover, the superimposition of the projected ROIs at their expected position can be viewed as an augmented reality technique that helps staff members to situate the structures of interest and to make sense of what they see.

There are some limitations to the method proposed in this work. The first one is the limited size of the EPID. Indeed, to avoid damaging the electronics of the detector, all treatments using beams with a field size larger than the detector are not eligible to this method. For this reason, when the projections are calculated with the ROI projection module before the treatments, each control point is verified in order to guarantee that the primary radiation field stays within the imaging area of the detector, with a small security margin, by using the leaves and jaws positions and the collimator angles contained in the DICOM RTPLAN. Another limitation is that the information is available in the beam's eye view only, which means that the position of the structures in the direction of the beam axis is not observable. The main effect of a small displacement in this direction would be a dose variation of a few percent in these structures due to a change to their distance relative to the radiation source and/or a change in their radiological depth. However, this would have much lesser clinical and dosimetric consequences than missing the target, which is why the beam's eye view geometry is preferable for 2D imaging.^21^ Also, as mentioned in the introduction, implantation of markers in the prostate is an invasive medical procedure and has associated risks. In our clinic, we already consider that the benefits of using markers for interfraction CBCT registration are sufficiently large compared to the risks of the procedure, so the software was used with patients who already have markers implanted.

One more limitation of the method is that the monitoring is limited to the moments where the leaves and jaws do not hide the structures the user is trying to locate. Nevertheless, our results showed that for prostate cancer patients treated using VMAT plans in our clinic, fiducial markers position can be verified at regular intervals throughout the entire duration of treatments. When the leaves or jaws do hide a structure of interest, the superimposed contours give the operator a visual cue that this structure is supposed to be hidden at that moment. Still, this interplay effect can cause a small delay before the therapist realizes that a motion of the structure occurred. Also, even with the automatic adjustment of image contrast using histogram equalization, there are still some cases where the images are not easily interpreted. In situations where only one marker is visible, there may be uncertainty in determining the contour to which the marker belongs. However, if the position of the markers changes slowly from one image to another, successive appearances and disappearances (behind the MLC) of a marker at the same location can help to identify the marker and its associated contour. When the displacement of the markers is large between images, the correlation between the trajectory of a marker and the trajectory of a contour can be used to identify the reference contour. Nevertheless, this uncertainty does not usually last long because it is common to see more than one marker (34% of the time on average according to Table 1). Finally, the iCom messages may not be the best way to correlate the gantry angles with the images. Indeed, the exact time at which the readout of the gantry angle is performed is not given with high precision in the messages. The gantry angle imprecision as well as the intrinsic resolution of the contours drawn on the images are the main limitations of the accuracy of the method but are small enough for our practical clinical use.

Despite the listed limitations, the proposed method contributes to improve the quality and safety of treatments. Preliminary clinical use of this method proved to be useful in our institution for VMAT treatments of prostate cancer with implanted fiducial markers. Indeed, prostate gland motion was frequently observed using this tool and in some cases the treatment beam was interrupted. Our criterion for interrupting a treatment was that at least one fiducial marker systematically touches or crosses over its tolerance contour during several successive images. Treatments were resumed after a short pause or, when necessary, by performing a CBCT and repositioning the patient. These results are not presented here since it is not in the scope of this study to characterize the movements of the prostate during treatments. We also believe that this tool can be useful when deciding which PTV margins are adequate. Because the tolerance margins for intrafraction motion are fixed by the planner and because the treatment can be manually stopped when the markers move outside those margins, the intrafraction motion contribution to the PTV margins equation can be fixed.^22^

This technique could also be applied to other treatment sites. For anatomical regions where a significant contrast exists between the target and the normal tissues, such as lung, the technique could potentially be used without fiducial markers. This would be especially useful for lung SBRT treatments as they are longer to deliver, therefore increasing the risk of the target getting out of the tolerance margins. The common practice of performing intrafraction CBCTs^23^ could be partially replaced by this technique. However, this site is proving to be challenging because the ability to discern the GTV from its surroundings depends on its density, its size, and its location.

## 5 CONCLUSION

We introduced a free open‐source software designed for intrafraction monitoring which uses projected ROIs contours in a beam's eye view geometry. We validated the accuracy of the technique and its clinical usefulness in a standard VMAT treatment delivery for prostate cancer patients with implanted fiducial markers. This technique has the major benefit of potentially improving patient care with very little upfront cost and no additional delivered dose.

## CONFLICT OF INTEREST

No conflicts of interest.

## Notes

^1^ Detector elements were binned 2 × 2 to account for GPU limitations. Therefore, the virtual EPID has 512 × 512 pixels instead of 1024 × 1024.

^2^ Here, we refer to the Elekta direction convention A‐B sides and Gun‐Target ends (GT) of the linear accelerator
